# Algorithmic fandom: how generative AI is reshaping sports marketing, fan engagement, and the integrity of sport

**DOI:** 10.3389/fspor.2025.1597444

**Published:** 2025-05-08

**Authors:** Hans Westerbeek

**Affiliations:** Victoria University Business School, Victoria University, Melbourne, Australia

**Keywords:** AI, artificial intelligence, AI integrity, algorithm, generative AI, data monetisation, echo chambers, fan engagement

## Abstract

Generative artificial intelligence (AI) is rapidly transforming the landscape of sports marketing by enabling hyper-personalised fan engagement, real-time content delivery, and data-driven commercial strategies. Leveraging technologies such as machine learning, predictive analytics, and automated content generation, sports organisations are increasingly able to tailor fan experiences, anticipate behaviour, and optimise revenue models. While these advancements offer significant opportunities for enhancing interactivity and commercial growth, they also introduce complex ethical, psychological, and regulatory challenges. This paper critically examines the dual nature of generative AI in sports marketing, with a particular focus on consumer autonomy, data monetisation, and the influence of AI-driven personalisation on fan behaviour. Using narrative literature review approach, the paper draws on emerging research, industry cases, and interdisciplinary literature, and explores how algorithmic recommendation systems can manipulate fan decisions, reinforce digital echo chambers, and marginalise underrepresented sports. Special attention is given to the impact of AI on children and adolescents, who are particularly vulnerable to targeted content, gamification, and AI-curated betting environments. The integration of AI in gambling platforms and the commercialisation of fan data raise significant concerns around privacy, consent, and long-term wellbeing. The paper concludes by offering some implementation guidance for sport ecosystem stakeholders. It also outlines a future research agenda that calls for empirical investigations into the long-term effects of AI on fan behaviour, the development of regulatory frameworks to safeguard consumer rights, and interdisciplinary collaboration to design ethical AI systems. By identifying these critical issues, the paper aims to support a more inclusive, transparent, and integrity-focused application of AI in sport.

## Introduction

The integration of generative artificial intelligence (AI) in sports marketing is fundamentally reshaping fan engagement by enabling hyper-personalisation and real-time content adaptation. AI-driven technologies, including machine learning, predictive analytics, and automated content generation, have introduced new avenues for enhancing fan experiences while optimising commercial strategies ( 1 ). These advancements are driving innovation in content delivery, audience segmentation, and behavioural prediction, contributing to the development of more immersive and interactive sports consumption models. Beyond its sport marketing applications, AI has also demonstrated utility in health promotion and training, where machine learning and deep learning approaches are being used to personalise physical activity programs across diverse populations ( 2 ). This signals the wider potential of AI as a tool for engagement and inclusion through sport and physical activity.

While these technologies present transformative possibilities for enhancing fan experiences and unlocking new business models, they also raise pressing questions about data security (3) consumer autonomy, data exploitation, algorithmic bias, and the integrity of engagement strategies—particularly in contexts involving children and vulnerable populations. The purpose of this paper is to critically explore these dualities: to examine how generative AI can deliver powerful commercial and experiential outcomes in sports marketing, while also surfacing the ethical, psychological, and regulatory challenges that accompany its deployment (4, 5).

### Narrative literature review

A narrative literature review methodology was adopted, selected for its flexibility in addressing an emerging an interdisciplinary topic—generative artificial intelligence (AI) in sport. A narrative review approach is well suited to synthesising diverse sources of knowledge across disciplines, including sport science, digital marketing, consumer psychology, and AI ethics. Unlike systematic reviews, narrative reviews allow the inclusion of both academic and non-academic sources, enabling a broader understanding of rapidly evolving technologies and their implications. Relevant literature was identified through targeted searches across multiple databases and platforms, including Frontiers; Wiley Online Library; SpringerLink; MDPI; Taylor & Francis; IEEE Xplore; arXiv.org; ResearchGate; and Google Scholar. Except for some foundational theoretical papers, the inclusion period focused on works published between 2022 and 2025, reflecting the post-ChatGPT surge in scholarly and industry interest in generative AI applications. Articles were included if they addressed AI-driven personalisation, algorithmic influence, data ethics, sport marketing, or digital fan engagement. The narrative review method supported the integration of peer-reviewed articles, preprints, government reports, and industry commentary. This was essential to critically explore the dualities of AI in sport—its commercial promise and its ethical, psychological, and regulatory challenges—particularly in areas such as youth engagement, gambling, and algorithmic bias.

Therefore, by synthesising emerging academic and industry insights, this paper aims to identify key issues in the use of AI for fan engagement and personalisation, and to propose a future research agenda that supports responsible innovation. In doing so, it contributes to a growing body of literature concerned with balancing technological advancement with the need to protect the trust, wellbeing, and diversity of sporting communities in the digital age.

### Conceptual foundation

The influence of generative AI on fan engagement is situated within a broader theoretical tradition drawn from sports marketing and consumer behaviour research. Foundational models such as Aaker's brand equity model (6), the hierarchy of effects model (7), and the expectancy-disconfirmation theory (8) offer enduring insights into how consumers form attitudes, make decisions, and develop loyalty. These models remain relevant as AI introduces new mechanisms for influencing behaviour, particularly through hyper-personalised content and predictive engagement tools. Within the field of sports marketing, frameworks that examine fan motivation and identification (9), sponsorship effectiveness (10), and consumer loyalty (11) provide essential grounding. Such work conceptualises fan engagement as an interplay between psychological attachment, motivational fulfilment, and consistent behavioural reinforcement. These are dynamics that AI systems now amplify through algorithmic curation and data-driven personalisation.

Recent research has begun bridging these traditional models with the realities of AI-enhanced environments. For example, Xu and Baghaei (12) demonstrate how generative AI is reshaping the contours of fan experience through dynamic storytelling and emotion-driven interaction, but they also highlight that issues in regard to data privacy, algorithmic bias and fair competition need to be addressed. IBM's (13) global fan study identifies AI as a critical enabler of real-time, personalised content that can both deepen loyalty and commercialise emotional touchpoints. These developments support a hybrid conceptual model, in which legacy theories of brand attachment and motivational satisfaction are reinterpreted in light of real-time algorithmic feedback loops and hyper-responsive engagement systems.

In parallel, the regulatory context of AI adoption in sport must be seen through a global lens. As will be outlined later in this paper, while Australia has introduced restrictions around AI-powered gambling content targeting children (14, 15), other jurisdictions are shaping different policy landscapes. For example, the European Union's AI Act, implemented in 2024, classifies AI applications in biometric surveillance (very relevant to sport business) as “high risk” and subject to stricter oversight (16). In contrast, the United States has taken a sector-specific approach, with emphasis on transparency, accountability, and the role of self-regulation (17). Canada, similarly, has pursued legislation under the Artificial Intelligence and Data Act (AIDA), which introduces risk-based obligations for AI deployment (18). Understanding how socio-political contexts shape AI regulation is essential to assessing its long-term viability and integrity in sport. For instance, the European emphasis on rights-based regulation may limit the commercial freedoms of AI-curated fan engagement platforms, while the more permissive U.S. environment could encourage rapid innovation but exacerbate risks related to data privacy and algorithmic discrimination.

By integrating these historical and contemporary theoretical perspectives and accounting for diverse regulatory regimes, this article situates generative AI not merely as a technological development, but as a transformative force embedded in complex behavioural, ethical, and socio-political systems. We will now first turn our attention to the sport consumers.

### Fan decision-making

The influence of AI extends beyond content generation to shaping consumer decision-making through targeted advertising, predictive analytics, and algorithmically driven personalisation strategies (19). AI can anticipate a fan's preferences based on historical data, browsing habits, and engagement patterns, refining marketing messages to optimise conversion rates (20). This approach has proven highly effective in driving ticket sales, merchandise purchases, and digital subscriptions, yet it also raises concerns about the extent to which AI influences fan autonomy. Fans may believe they are making independent choices when, in reality, their options are being carefully curated by technology designed to maximise engagement and spending.

The ethical implications of AI-driven decision-making are particularly relevant in the context of younger fans, to be tackled in more detail later. Young people are more susceptible to digital nudging and personalised marketing ( 21 ). AI-generated content often leverages behavioural psychology to encourage engagement and spending, from gamified reward systems in sports apps to AI-driven loyalty programmes that incentivise continued participation. While these techniques enhance fan engagement, they also raise concerns about the long-term impact of AI on how much the consumer still is control of his or her own consumption destiny.

### Consumer autonomy

One of the primary concerns associated with AI-driven personalisation in sports marketing therefore is the potential erosion of consumer autonomy. AI systems, designed to maximise engagement and revenue, rely on vast amounts of consumer data to refine marketing strategies, often guiding purchasing decisions and content consumption in ways that may not be immediately transparent to the individual ( 22 ). These concerns mirror findings in elite sport settings, where explainability, data integration, and human oversight remain key challenges to AI adoption ( 4 ). In sports science, where AI may support performance indicators such as load management and injury prevention, the most effective use of AI is when it complements rather than replaces expert judgement ( 23 ). AI in that regard should complement rather than drive consumer choice in its role in fan engagement. While personalisation is often framed as an enhancement of user experience, the extent to which AI influences consumer choice raises fundamental ethical concerns about agency, decision-making freedom, and the long-term implications of AI-mediated interactions.

Making fans feel that the sport organisation or its athletes know them personally and are interested in their lives and sporting passions, can be achieved by operating sophisticated behavioural prediction models that analyse past interactions to determine future preferences. These models are not merely reactive but proactively shape and deliver on consumer choices. Such models provide options that align with predicted behaviours while subtly discouraging alternative choices ( 3 ). For instance, AI-powered ticketing platforms may steer consumers toward higher-tier seating options or bundled merchandise packages based on past purchases, nudging them toward spending patterns that maximise revenue. While consumers retain the ability to make final decisions, their choices are increasingly framed within algorithmically generated pathways that limit true independence in decision-making.

By continuously reinforcing existing preferences, these platforms may also reduce the diversity of content available to consumers, restricting exposure to alternative viewpoints, sports, or teams (24). This is particularly concerning in contexts where AI prioritises engagement metrics over variety, leading to a narrowing of consumer experience. A sports fan who predominantly engages with men's football content, for example, may find it increasingly difficult to discover women's sport or emerging leagues, as AI systems optimise content feeds to align with historical consumption patterns.

The challenge of distinguishing between personalisation and manipulation is further compounded by AI's ability to exploit cognitive biases. Psychological research suggests that consumers are more likely to trust recommendations that appear personalised, even if they are algorithmically generated (21). One such example is the confirmation bias where fans are more likely to engage with content that aligns with their existing beliefs or preferences. AI systems can reinforce this by serving similar types of content repeatedly—such as favouring highlights of a specific team or player a fan has shown interest in—thereby narrowing their exposure and subtly shaping their perceptions and future behaviours. This can be further leveraged to drive purchasing behaviours through AI-generated urgency prompts, such as limited-time offers or scarcity-based incentives. While these tactics have long been used in traditional marketing, AI enhances their effectiveness by tailoring them to individual behavioural patterns, making it more difficult for consumers to recognise when they are being influenced.

Furthermore, the increasing use of AI in customer service automation can also diminish consumer agency. AI chatbots and virtual assistants, designed to handle fan inquiries and facilitate transactions, often present predefined options rather than open-ended solutions, subtly guiding users toward predetermined choices that align with organisational goals ( 1 ). This structured engagement model, while efficient, reduces the scope for consumer-driven exploration and decision-making, reinforcing the influence of AI in shaping consumption behaviours. Of course, in any type of business including sport, one of the key foci is on profit maximisation. Algorithms will be programmed to make money from the data that they transform.

### Data monetisation

The commercialisation of fan data therefore represents both a lucrative opportunity and another ethical challenge. AI-powered analytics allow organisations to refine pricing models, optimise sponsorship activations, and develop targeted advertising strategies that optimise revenue streams ( 1 ). By leveraging machine learning algorithms to analyse vast datasets, sports organisations can gain unprecedented insights into fan behaviour, enabling them to predict purchasing tendencies. Those insights can be used to personalise engagement strategies and increase the return on investment for sponsors. However, as AI-driven data monetisation accelerates, concerns regarding consumer privacy, ethical data use, and regulatory oversight are becoming increasingly critical ( 3 ).

A significant development in sports marketing is the use of dynamic pricing models, where ticket prices fluctuate based on real-time demand, individual purchasing histories, and broader market trends. While this strategy maximises profitability, it raises ethical concerns about fairness and transparency. AI-powered ticketing platforms, for instance, may use behavioural data to personalise pricing, offering different price points based on a fan's likelihood to purchase. While some consumers may benefit from discounted rates, others may find themselves paying higher prices based on predictive analytics, leading to questions about price discrimination and data ethics (22).

The growing reliance on AI-driven data monetisation also has implications for sponsorship models. Traditionally, sponsorship effectiveness was measured through broad audience demographics, but AI now enables micro-targeting, ensuring that advertisements reach specific fan segments based on engagement patterns ( 19 ). For example, a fan who frequently engages with social media content related to a particular club or athlete may be targeted with AI-generated, hyper-personalised sponsorship messages that seamlessly integrate with their browsing experience. While this enhances marketing efficiency, it also highlights how AI systems can manipulate user attention and by doing that, reinforce commercial dependencies.

Beyond ticketing and sponsorship, AI-driven data monetisation has expanded into developing new revenue streams. One rapid-growth area is AI-powered sports betting platforms, which utilise fan engagement data to personalise betting recommendations and promotions. This integration between sports marketing and gambling present new challenges. Later in this paper this will be discussed in particular reference to younger fans who may be unknowingly nudged toward betting behaviours through AI-curated content and targeted advertisements. The ability of AI to predict betting patterns and personalise gambling incentives increases the risk of addiction, prompting calls for stricter regulatory frameworks to prevent exploitative practices ( 25 ).

The resale and exchange of consumer data among third-party stakeholders also is a double-sided coin of opportunity and threat. Many sports organisations partner with external brands, digital platforms, and analytics firms, leading to complex data-sharing ecosystems. While these collaborations can enhance marketing precision and drive commercial growth, they also pose risks regarding data security and consumer consent ( 26 ). Studies indicate that many fans remain unaware of how their personal data is collected, stored, and used, due to the lack of transparency of AI-driven data ecosystems ( 3 ). The increasing prevalence of AI-powered surveillance and tracking technologies further complicates the ethical landscape, as organisations gather granular data on fan movements, online interactions, and purchasing habits. Research from related domains also shows that trust and commitment to digital service interfaces are closely tied to data security practices, especially when robotic or AI-driven systems mediate the customer experience ( 5 ).

### Funnelling consumption down the rabbit hole

As briefly introduced earlier, the extent to which these technologies shape and narrow consumer behaviour by reinforcing existing consumption patterns will make for an inferior consumption experience. It was already noted that delivering content that aligns with a user's past interactions, will gradually limit exposure to alternative perspectives, sports, and athletes (24). While this enhances personalisation and strengthens fan retention, it also creates digital echo chambers where consumers are repeatedly exposed to the same type of content, reducing diversity in sports media consumption. This phenomenon has significant implications for both fan experience and the commercial landscape of sport, as certain teams, leagues, and athletes gain disproportionate visibility at the expense of others. Those sport organisations or athletes most digitally present will accelerate their popularity and earning power while the less visible contenders will increasingly fall behind and lose competitiveness.

This will lead to marginalised and underrepresented sports and athletes. The dominance of mainstream sports in AI-generated content feeds often results in a self-reinforcing cycle: the more a fan interacts with high-profile teams and athletes, the more the algorithm promotes similar content, further reducing exposure to emerging sports or less commercially viable leagues (25). This is particularly evident in the disparity between men's and women's sports coverage, where algorithms, driven by historical engagement data, continue to prioritise men's competitions, perpetuating long-standing visibility gaps. If left unchecked, AI's role in funnelling consumption toward already dominant sports properties could undermine efforts to promote equity and inclusivity in the sports industry.

Beyond limiting content diversity, AI-generated engagement also raises concerns about misinformation and the authenticity of digital narratives. Generative AI tools have been found to produce “hallucinations”—outputs that appear credible but are factually incorrect or misleading (25). In the context of sports media, this can manifest in several ways, including AI-generated match reports that misrepresent events, misleading player statistics, or algorithmically curated news feeds that amplify sensational or misleading narratives. Given that sports fans often rely on digital platforms for real-time updates, the risk of misinformation being widely disseminated before it can be corrected is a growing concern. This “fake” or dark-side nature of generative AI has been further underscored in cybersecurity research, where tools like ChatGPT can be manipulated to craft malicious content or bypass privacy safeguards, raising serious implications for how fan data is stored and protected (27).

### Dangers of generative AI in decision-making among children and adolescents

Yuan et al. (28) caution against overreliance on generative AI in educational and behavioural contexts, noting the risk of diminished critical thinking and originality—a concern equally pertinent to how children engage with AI-curated sports content. As AI-powered systems become more sophisticated in predicting consumer behaviour and tailoring content to individual preferences, younger audiences—who are more impressionable and less equipped to critically evaluate algorithmically curated experiences—are particularly vulnerable to persuasive digital environments (19). Research in consumer psychology also suggests that adolescents are more susceptible to AI-driven nudging techniques due to their developing cognitive and decision-making capacities, making them an attractive target for AI-enhanced marketing strategies (21). For example, sports streaming services that employ AI to suggest premium content upgrades may encourage impulsive spending, capitalising on behavioural tendencies associated with instant gratification, access and exclusivity.

As briefly noted earlier, the role of AI-driven gambling and sports betting platforms in shaping the behaviours of young consumers is a matter of great concern. Digital gambling companies have increasingly embedded AI in their engagement strategies, using machine learning to predict betting habits, personalise incentives, and optimise user retention ( 3 ). The intersection of AI, sports media, and gambling is particularly troubling, as it exposes young fans to betting-related content through in-game advertisements, predictive analytics, and real-time odds recommendations. The ability of AI to personalise these experiences means that young users who demonstrate early engagement with betting-related content may find themselves in an algorithmically curated environment that continuously reinforces gambling behaviours ( 25 ). What is the duty of sports organisations and digital platforms to safeguard vulnerable audiences from the long-term risks of AI-driven gambling exposure?

For example, in Australia, the government has implemented several legislative measures to mitigate the impact of sports betting advertising on children, aiming to protect young audiences from exposure to gambling promotions. In March 2018, regulations were introduced to prohibit gambling advertisements during live sports broadcasts between 5:00 am and 8:30 pm. This measure was designed to reduce children's exposure to betting promotions during times when they are likely to be watching sports events (15). The Australian federal government has been preparing to introduce legislation aimed at further restricting the amount and timing of gambling advertisements. Specifically, the proposed reforms would see gambling advertising banned in the hour before and after live sport, with a new cap of two ads per hour on free-to-air TV until 10:00 pm (29). Additionally, there is a proposal to ban gambling ads during children's programming and live sports broadcasts, and to cap ads in other slots at two per hour (14). These measures are intended to reduce the exposure of children and vulnerable audiences to gambling promotions.

In reference to earlier mentioned digital echo chambers that reinforce specific interests and behaviours, young fans may be exposed to highly targeted content streams that limit their exposure to diverse sporting narratives and experiences. This concern is particularly relevant in relation to gender representation in sports media, as AI-driven content selection may perpetuate existing disparities by prioritising content based on historical engagement patterns rather than promoting inclusivity ( 24 ). The narrowing of content diversity not only impacts sports consumption of young people, but also shapes long-term perceptions of which sports, teams, and athletes are deemed significant.

A final observation relates to the potential for AI-driven content generation to introduce misinformation and deceptive marketing tactics. Generative AI tools can create highly realistic but misleading narratives, making it difficult for young audiences to distinguish between authentic and AI-generated content ( 25 ). This issue is particularly pertinent in environments where AI is used to craft promotional messages, endorsements, and influencer-style content, as young users are more likely to trust and engage with AI-generated media that mimics familiar and authoritative sources ( 19 ). Without proper regulatory oversight and transparency in AI-generated marketing, there is a risk that young consumers will develop skewed perceptions of reality, further complicating their decision-making processes.

## Discussion

Generative AI is no longer a speculative technology—it is actively reshaping the architecture of sports marketing, transforming how fans interact with content, make consumption decisions, and engage with their favourite teams and athletes. The commercial logic is clear: AI offers unprecedented opportunities for hyper-personalised engagement, behavioural prediction, and revenue optimisation (1, 19). However, the same technological mechanisms that power these innovations also raise fundamental questions about autonomy, equity, and integrity in sport marketing ecosystems.

This paper has examined how AI-driven recommendation systems, dynamic pricing algorithms, and predictive engagement tools are not only shaping what fans consume, but also how they think, feel, and act in sport environments. These systems exploit well-documented cognitive biases, such as confirmation bias and trust in personalisation (21), narrowing content diversity and reinforcing digital echo chambers (24). For underrepresented sports, women's competitions, and emerging markets, this risks perpetuating systemic visibility gaps and algorithmic exclusion.

The monetisation of fan data is another axis of concern. While AI analytics allow for granular targeting and sponsorship precision, they also expose consumers to opaque data practices, commercial surveillance, and uneven privacy protections (3, 26). These dynamics are particularly problematic when applied to children and adolescents, who are more vulnerable to persuasive AI environments, gamified rewards, and AI-curated betting content (25, 28). The intersection of AI and sports gambling raises the stakes considerably, with early exposure to algorithmically tailored betting promotions posing significant risks to young consumers' long-term wellbeing. Importantly, these are not just ethical abstractions—they are practical challenges that require urgent empirical investigation. As noted by Gupta et al. (27), generative AI systems can also be weaponised, introducing misinformation, security vulnerabilities, and data misuse into sports media ecosystems. This further complicates the sometimes fragile trust between fans and sport institutions.

To address these risks responsibly, stakeholders across the sport ecosystem such as sport organisations, sponsors, technology partners, regulators, educators, and fans, must adopt a more proactive stance. This includes operationalising ethical oversight mechanisms and building long-term digital resilience among users. For example, ongoing algorithmic audits should be mandated or voluntarily adopted by sports organisations and their digital partners. These audits conducted by internal teams or independent third parties can help detect bias in content curation (such as overrepresentation of men's sport), identify manipulative patterns in personalised pricing or gamified engagement, and ensure compliance with data governance regulations such as the EU AI Act (16). Such audits could also become a cornerstone of sport integrity frameworks. Alongside technological oversight, targeted media and digital literacy initiatives should be developed, particularly for children, adolescents, and vulnerable audiences. These initiatives should equip individuals to recognise biased or overly personalised content; actively challenge algorithmic recommendations by seeking diverse sporting narratives and underrepresented voices; and understand the terms of service and data practices of the platforms they engage with. Embedding this content into educational curricula, sport club programs, and parental guidance tools would foster greater autonomy and reduce the risk of manipulation.

In light of these issues, future research should prioritise the development of evidence-based frameworks that interrogate the long-term behavioural, social, and psychological effects of AI-driven sports marketing. First, longitudinal studies are needed to assess how AI-powered personalisation impacts fan loyalty, spending behaviour, and content diversity over time. Second, research should explore mechanisms to mitigate algorithmic bias in content recommendation systems, ensuring more equitable visibility for diverse sports, athletes, and narratives. Third, there is a pressing need to evaluate the effectiveness of regulatory interventions—such as advertising restrictions, data transparency mandates, and age-specific safeguards—within the context of AI-enhanced sport betting and personalised promotions (14, 15, 17, 18, 29).

Moreover, stakeholders such as sponsors and advertisers should take an active role in ensuring ethical use of AI in fan engagement. Sponsors can demand transparency from sport and tech partners, promote ethical codes for AI-assisted marketing, and avoid practices that target vulnerable users with betting or high-pressure sales content.

Finally, interdisciplinary collaboration between sport marketers, data scientists, behavioural scientists, and policymakers will be essential. Ethical AI governance in sport cannot rely on technical solutions alone; it must be informed by a nuanced understanding of how fans interpret, resist, and adapt to AI-curated environments. The goal is not to slow innovation, but to shape it—ensuring that the sport industry remains a space of fair play, inclusivity, and trust, even as it becomes increasingly algorithmic. By embedding these research priorities into academic and industry agendas, we can help ensure that generative AI becomes a force not only for commercial growth, but for ethical advancement and integrity in global sport.

## Data Availability

The original contributions presented in the study are included in the article/Supplementary Material, further inquiries can be directed to the corresponding author.
